# Prevalence and Comorbidities of Attention Deficit Hyperactivity Disorder Among Adults and Children/Adolescents in Korea

**DOI:** 10.1192/j.eurpsy.2023.1574

**Published:** 2023-07-19

**Authors:** W.-M. Bahk, J. S. Seo, H.-M. Sung, S.-Y. Park

**Affiliations:** 1 Psychiatry, The Catholic University of Korea, Seou; 2 Psychiatry, Chung-Ang University Hospital, Gwangmyeong; 3 Psychiatry, Soonchunhyang University, Gumi; 4 Psychiatry, Keyo Hospital, Uiwang, Korea, Republic Of

## Abstract

**Introduction: Objectives:**

This study investigated the prevalence and comorbidities of attention deficit hyperactivity disorder (ADHD) among adults and children/adolescents in Korea.

**Methods:**

This study used data from the Korea Health Insurance Review and Assessment Service collected from 2008 to 2018. Study participants comprised patients with at least one diagnosis of ADHD (International Statistical Classification of Diseases and Related Health Provisions, 10th revision code F90.0). Prevalence rates and psychiatric comorbidities were also analyzed.

**Results:**

We identified 878,996 patients diagnosed with ADHD between 2008 and 2018. The overall prevalence rate of diagnosed ADHD increased steeply from 127.1/100,000 in 2008 to 192.9/100,000 in 2018; it increased 1.47 times in children/adolescents (≤ 18 years) and 10.1 times in adults (> 18 years) during this period. Among children/adolescent and adult ADHD patients, 61.84% (95% confidence interval [95% CI] 61.74−61.93) and 78.72% (95% CI 78.53−78.91) had at least one psychiatric comorbidity, respectively.

**Conclusions:**

Our results showed that the prevalence rate of diagnosed ADHD has increased in Korea; however, it is lower than the global average. Further studies are required to identify and treat vulnerable populations appropriately.

**Disclosure of Interest:**

None Declared

